# A Network Approach of Gene Co-expression in the *Zea mays*/*Aspergillus flavus* Pathosystem to Map Host/Pathogen Interaction Pathways

**DOI:** 10.3389/fgene.2016.00206

**Published:** 2016-11-21

**Authors:** Bryan M. Musungu, Deepak Bhatnagar, Robert L. Brown, Gary A. Payne, Greg OBrian, Ahmad M. Fakhoury, Matt Geisler

**Affiliations:** ^1^Department of Plant Biology, Southern Illinois University, CarbondaleIL, USA; ^2^Warm Water Aquaculture Unit, United States Department of Agriculture – Agricultural Research Service, StonevilleMS, USA; ^3^Southern Regional Research Center, United States Department of Agriculture – Agricultural Research Service, New OrleansLA, USA; ^4^Department of Plant Pathology, North Carolina State University, RaleighNC, USA; ^5^Department of Plant Soil and Agriculture Systems, Southern Illinois University, CarbondaleIL, USA

**Keywords:** *Aspergillus flavus*, Zea mays, networks, RNA-sequencing, gene co-expression analysis

## Abstract

A gene co-expression network (GEN) was generated using a dual RNA-seq study with the fungal pathogen *Aspergillus flavus* and its plant host *Zea mays* during the initial 3 days of infection. The analysis deciphered novel pathways and mapped genes of interest in both organisms during the infection. This network revealed a high degree of connectivity in many of the previously recognized pathways in *Z. mays* such as jasmonic acid, ethylene, and reactive oxygen species (ROS). For the pathogen *A. flavus*, a link between aflatoxin production and vesicular transport was identified within the network. There was significant interspecies correlation of expression between *Z. mays* and *A. flavus* for a subset of 104 *Z. mays*, and 1942 *A. flavus* genes. This resulted in an interspecies subnetwork enriched in multiple *Z. mays* genes involved in the production of ROS. In addition to the ROS from *Z. mays*, there was enrichment in the vesicular transport pathways and the aflatoxin pathway for *A. flavus*. Included in these genes, a key aflatoxin cluster regulator, AflS, was found to be co-regulated with multiple *Z. mays* ROS producing genes within the network, suggesting AflS may be monitoring host ROS levels. The entire GEN for both host and pathogen, and the subset of interspecies correlations, is presented as a tool for hypothesis generation and discovery for events in the early stages of fungal infection of *Z. mays* by *A. flavus*.

## Introduction

A systems biology approach can greatly aid in understanding the dynamics of fast changing systems like stress and defense responses in plants, and allows for both discovery and novel hypotheses generation. The rise of the systems biology field has been brought on by the recent expansion of genomic, transcriptomic, and proteomic technologies and their large comprehensive datasets. “Omics" based techniques, such as gene co-expression networks (GENs), have aided in the discovery and functional analysis of genes and pathways involved in the response to biotic and abiotic stresses in plants (Sekhon et al., 2013). Gene expression analysis has been done individually on plants and fungi such as *Zea mays*, *Glycine max* (Libault et al., 2010), *Arabidopsis thaliana* (Mao et al., 2009), and *Saccharomyces cerevisiae* (Carlson et al., 2006).

In studies of host–pathogen interactions, however, two key limiting factors of system biology approaches involving genome-wide transcriptional studies have been typically observed. First, co-expression networks, especially those that have relied on microarrays, have been limited by the size of the array used to generate the data, which can lead to a loss in information. Secondly, the more extensive RNA-sequencing based studies thus far have often been limited to either the host or the pathogen.

The opportunistic pathogen *Aspergillus flavus* produces the potent mycotoxin aflatoxin upon infecting of *Z. mays*. This interaction was chosen to study because the pathogen has been reported to transition from a saprophytic to a necrotrophic lifestyle during the infection process (Abdel-Hadi et al., 2012; Fountain et al., 2014). Moreover, *A. flavus* typically infects maize kernels at a specific stage of maize development (Diener et al., 1987). A transcriptomic network study of this host–pathogen interaction is thus a good complement to other developmental RNA-seq based co-expression network studies in maize and *A. flavus* (Sekhon et al., 2013; Schaefer et al., 2014). Additionally, the *A. flavus* GEN represents the first GEN published for *A. flavus*.

What is currently known about *A. flavus* and *Z. mays* has been primarily derived though a focus on either the host or the pathogen. These approaches have been beneficial and have resulted in the discovery and characterization of multiple secondary metabolite clusters in *A. flavus* (Kale et al., 2008; Chanda et al., 2010; Chang et al., 2013; Rohlfs, 2015). Examples of this have been in the transcriptome analysis of clusters such as the aflatoxin, aflatrem, and cyclapanozic acid (Chang et al., 1995, 2004; Ehrlich et al., 1999; Duran et al., 2007; Kale et al., 2008; Hong et al., 2015). These whole transcriptome studies strongly complemented bottom up experimental approaches, and filled in gaps toward a better understanding of these secondary metabolite pathways.

In *Z. mays*, multiple pathways have been discovered to be involved in resistance to *A. flavus*. In fact, pathways such as the phenylpropanoid, jasmonic acid, and salicylic acid pathways have been reported to play a significant role in resistance to the fungus (Warburton et al., 2015). Moreover, genome-wide association studies (GWASs) have indicated that the jasmonic acid pathway plays the largest role in conferring resistance across the current publically available breeding studies involving maize lines resistant to *A. flavus* (Tang et al., 2015; Warburton et al., 2015).

In this dual transcriptomic study of *A. flavus* and *Z. mays*, our focus was to infer a GEN of both host and pathogen during the initial stages of infection, and to develop a tool that could be utilized to understand the complex interaction between *A. flavus* and *Z. mays*. The approach used in this study differs from what has been previously reported in that the correlation of expression between genes in host and pathogen (cross-species) is captured along with correlations restricted to the host or the pathogen. The network approach used reveals features that are lost, particularly when relying on co-expression analysis using only heat maps and gene clustering. This work shows that there is significant cross-species co-expression during the initial stages of infection. It also reveals resistance and virulence mechanisms, particular to *A. flavus* and *Z. mays*, which are important at the initial stages of the interaction between the two organisms. The generated network maps can also serve as a tool to infer functional annotation for poorly annotated genes in both *Z. mays* and *A. flavus*.

## Materials and Methods

### Growth and Inoculation of Maize

Maize B73 was grown in Clayton, NC during the years 2011 and 2013. Seeds were planted on April 16. Ears were hand pollinated from July 5–8 and covered with a paper bag. A time course study was performed by pin bar inoculating one ear (per time point) of maize B73 with *A. flavus* NR3357 and harvesting at 0, 6, 12, 18, 24, 30, 36, 42, 48, and 72 h post-inoculation. Four biological replicates for the samples were produced for the time points 12, 24, 48, and 72 h post-inoculation. Samples were frozen in liquid nitrogen, placed on dry ice and stored at –80°C until RNA was isolated. Maize B73 and *A. flavus* NR3357 were used in this study because of the availability of genomic resources for them, and because of the relatively large amount of studies that were previously conducted using either or both of them. Maize B73 and *A. flavus* NR3357 were therefore used in this study to make comparisons to other available experimental data sets more straightforward.

### RNA Isolation

Eight kernels per ear were ground and used for RNA isolation. Approximately, 100 mg of ground tissue was homogenized in a Virtis homogenizer (Virtis, Gardiner, NY, USA) in Saturated Phenol, pH 6.6 for 2 min. Samples were then dissolved in Tris EDTA buffer, pH 8.0 (ACROS Organics, Morris Plains, NJ, USA), purified with 5:1 Acid Phenol: Chloroform, pH 4.5, and precipitated with ice-cold 100% ethanol overnight. Total RNA was purified again with RNeasy Mini Kit (Qiagen, Hilden, Germany) according to the manufacturer’s instructions. The quality and concentration of RNA was analyzed using an RNA Pico chip on an Agilent Bioanalyzer (Agilent, Santa Clara, CA, USA).

### Sequencing and Mapping

cDNA library construction and sequencing was performed at the Genomic Sciences Laboratory, North Carolina State University. Libraries were made from each time point, pooled and run on a single lane. Sequencing was performed on an Illumina HiSeq (single reads, 100 bp). Raw reads were deposited in the Sequence Read Archive (SRA) at NCBI, accession numbers [SRP082421]. Mapping, trimming and fastqc quality control of the reads, for both *Z. mays* and *A. flavus*, were done with CLC workbench 4.9 (Qiagen, Hilden, Germany). CLC genomics workbench default parameters were used for doing the mapping and trimming. The reference genomes used in this study where *Z. mays* (AGPv3, INSDC Assembly GCA_000005005.5, April 2013) and *A. flavus* (JCVI-afl1-v2.0, INSDC Assembly GCA_000006275.1, January 2009). Reads that had a total unique gene count of 1 were removed from the counts table.

### Gene Ontology Analysis

Gene ontology analysis of the co-expression subnetwork was done with the gprofiler and significant go-terms were selected based upon a false discovery rate (FDR <0.05) (Reimand et al., 2016).

### Regularized Log Normalization, M-Value Calculation, and K-Means Clustering

Unique mapped read counts were normalized by regularized log transformation using the DESeq2 package in the R software environment (ver. 3.3). The mapped reads were than used to calculate M-values for the different samples by using samples 0 and 6 h as controls for Dataset 1 (Love et al., 2014).

$$\left[M-value=\log_{2}\left(\frac{treatment}{control}\right)\right]$$

The DESeq2 function plotPCA() was used to generate principal component plots to analyze the variation in the samples. For *Z. mays*, mRNA extracted from samples collected 0 hpi were used as control. As for *A. flavus*, samples collected 24 hpi were used as control. The original matrix contains 7408 genes (3331 *Z. mays* and 4115 *A. flavus*) showed significant differential expression with a q-value cutoff <0.1 (after Benjamini Hochberg FDR correction) and were selected for clustering and gene co-expression analysis. After duplicate gene models were removed the matrix was reduced to a size of 6035 genes (*A. flavus* 3290 and *Z. mays* 2745). TM4 (Saeed et al., 2006) and Cluster 3.0 (de Hoon et al., 2004) software platforms were used to analyze these genes by K-means clustering using the absolute Pearson correlation metric and average cluster sampling method into two different bin sizes (10, 100) (**Supplementary Table S1**).

### Correlation Calculation and Network Analysis

Pearson correlation analysis was done on differentially expressed genes by utilizing the cor() function in R using the Z-scores calculated (see above). The resulting matrix was then transformed into a pair-wise gene table using the reshape2 melt() package in R. The resulting table (∼30 million gene pairs) was filtered using the subset() function in R for gene pairs showing 0.95 or greater correlation. This resulted in 980,280 gene pairs. In addition a randomly generated matrix was done similarly for the data which was obtained generating random log2Fold changes were generated in R for subsequent Kolmogorov–Smirnov supplemental test (Massey, 1951).

### Network Visualization and Cytoscape

To visualize the network 980280 gene pairs were load into the cytoscape network. Gene Annotation was then added to the network using the (AGPv3, INSDC Assembly GCA_000005005.5, April 2013) and *A. flavus* (JCVI-afl1-v2.0, INSDC Assembly GCA_000006275.1, January 2009). It is important to note that the best method for visualizing the network is to import the data into Cytoscape without building the network if not using a large memory computer. To select gene pairs of interest the user can refer to the Cytoscape manual in order to select for genes of interest and first neighbors without a high memory PC. Network topological analysis was then done on this set of gene pairs using Cytoscape Ver. 3.2.1 (Shannon et al., 2003; Maere et al., 2005; Cline et al., 2007). The network analysis was performed using the NetAnalyzer tool in Cytoscape and network clusters were identified using the MCODE app for Cytoscape (Bader and Hogue, 2003).

## Results

### RNA-seq

RNA-seq was carried out on mRNA extracted from *Z. mays* kernels inoculated with *A. flavus* over a 3-day time course. The individual reads were competitively mapped to a combined gene model set of both *A. flavus* (13775 gene models) and *Z. mays* (181929 gene models). 44264 gene models had unique mapped reads in at least one pooled kernel sample, while 151440 gene models did not have any expression (0 unique mapped reads) across all the samples. 6035 genes were then selected for co-expression analysis based upon their significant level of differential expression (see Materials and Methods). The *A. flavus* 3290 and *Z. mays* 2745 genes are stored in a matrix (Supplemental File 1).

### Principal Component and K-Means Clustering Analyses

**FIGURE 1:**
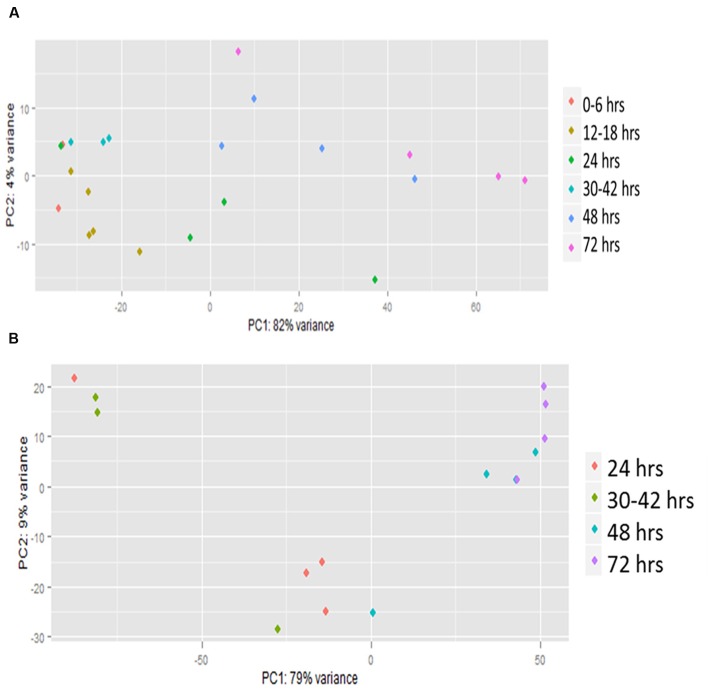
**Principal component analysis (PCA) of RNA-seq.** Transcriptomic (global gene expression) data from each of the time points of **(A)** *Zea mays* and **(B)** *Aspergillus flavus* were analyzed by principal component analysis using R statistical language. Colored dots represent individual biological replicates harvested at different time points during the infection. These were grouped based on similarity of infection by hpi (hours post-inoculation).

Principal component analysis (PCA) showed a high level of variation between samples, but many of the later time points clustered together (**Figure 1**). The analysis also showed that a high level of variation occurred between samples collected at the same hpi (hours post-infection) for both host and pathogen (**Figures 1A,B**). When visualized using centroid graphs from K-means clustering to observe expression patterns there was a high level of variation between *Z. mays* and *A. flavus* at 12 hpi (hours post-infection), 48 hpi, and 72 hpi for genes in both systems. These observations lead us to conclude that although these were indeed biological replicates, inoculated with the same culture of *A. flavus*, and harvested for RNA at the same time, the progression of the infection could be quite different from replicate to replicate. These replicates thus likely represent leading and lagging infection progress at early and middle stages of infection. There is tight replicate grouping in early time points (**Figure 1A**, 12–18 hpi) and clear separation of these from later stages of infection (48–72 hpi). The pathogen *A. flavus* (**Figure 1B**), seems to have a more variable expression in early infection stages, but these have caught up to each other and give a more consistent expression profile at 72 hpi. For this reason, we treated each replicate as a separate data point (representing a different stage of infection), rather than using replicate averages in downstream clustering and correlation analyses. When the genes were clustered into 10 bins (clustering groups), the largest bin had 2599 correlated genes for *Z. mays* and for *A. flavus*. This largest cluster had a similar overall trend of down-regulation over time. Functional analysis of this cluster found genes enriched in multiple biological processes with no obvious pattern related to resistance to pathogens. Examples included response to cadmium ion, response to inorganic substance, protein folding, DNA replication, glucose homeostasis, and nucleosome assembly. Other bins contained smaller numbers of genes but produced similar results for functional enrichment.

Genes were then K-means clustered into 100 bins to determine functional enrichment of tightly inter-correlated groups of *A. flavus* and *Z. mays* genes (**Supplementary Table S2**). These bins had a smaller number of genes (3–1488) and many included clusters with genes from a single species. Nineteen clusters contained genes from both species. In many cases, genes related to disease resistance in *Z. mays*, even those with somewhat different biological roles (i.e., multi-domain genes and resistance to different pathogen types), appeared in the same cluster. For example, cluster 21 (from **Supplementary Table S2**) contained multiple primary metabolism and signaling kinase genes such as 3-Deoxy-Darabinoheptulosonate 7-phosphate (DAHP) synthase (GRMZM2G396212) and Inositol 1 3 4 trisphosphate 5/6-kinase family protein (GRMZM2G084609). Another cluster contained the genes encoding for SPP2 Sucrose-phosphatase 2 (GRMZM2G097641) and LOX10 lipoxygenase-10 (GRMZM2G015419). These genes were found to be co-expressed with multiple heat shock genes and with myb transcription factors. Many of the genes in this cluster were noted in previous studies involving resistance of maize to *A. flavus* and *Fusarium verticillioides*. An example is the presence in our dataset of WRKY19 (GRMZM2G063880), WRKY53 (GRMZM2G012724), and PR10.1 in *Z. mays* which have been previously shown to be up-regulated upon infection by *A. flavus* (Fountain et al., 2015).

### Gene Co-expression Network

An alternative approach to K-means clustering is to examine all significant pairwise correlations between genes as a network graph. This overcomes limitations in arbitrary group number selection. It also assays specific correlations between genes relying on fitting genes based on the group average expression of a bin. Moreover, networks can reveal patterns of interrelationships between individual genes not readily apparent in other clustering methods, and thus provide a complementary analysis to what can be gaged from other analysis approaches.

**FIGURE 2:**
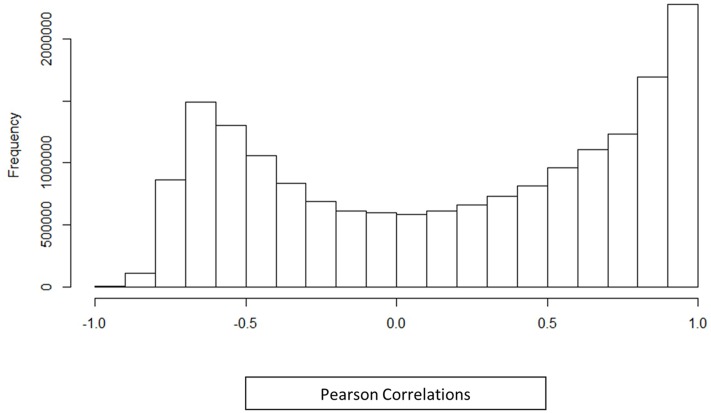
**Histogram distribution of Pearson correlations.** Histogram shows the correlation between differentially expressed genes identified in an *in vivo* simultaneous RNA-seq experiment with *A. flavus* and *Z. mays*. The histogram includes all differentially expressed genes along with their Pearson correlation distribution for (*Z. mays* vs. *Z. mays*), (*A. flavus* vs. *A.* flavus), and (*Z. mays* vs. *A. flavus*). The correlations that were found to be significant in the analysis were greater >0.95 Pearson correlation.

A co-expression network for the 6035 *Z. mays* and *A. flavus* differentially expressed genes was generated using pairwise Pearson correlation across all samples, and choosing an edge adjacency matrix with a cutoff of a (0.95) correlation coefficient. Surprisingly, even at this high correlation cutoff, the network was quite dense, and 980,280 significantly correlated gene pairs (edges) were discovered for 3638 genes (nodes). The distribution of the correlation values is shown in **Figure 2**. It can be seen from the distribution correlation values, that a majority of the correlations are skewed to the right (positive correlations) as has been shown in other co-expression analyses, including *Arabidopsis* and rice (Geisler-Lee et al., 2007; Ho et al., 2012). Further exploration of this network was to determine how similar the distribution was to a randomly generated distribution (see Materials and Methods). A Kolmogorov–Smirnov test showed a significant difference between the random and observed correlation value distributions (*p*-value 2.26 × 10^–16^) (**Supplemental Figure S1**). The observed distribution represents ∼1% of all the possible edges in the network. The high density is likely because we are looking at the small subset of differentially expressed genes involved in a single (kernel) tissue, subject to a large singular condition (pathogen attack) while most other similar GENs tend to involve all genes in multiple tissues and different stresses in their analysis. The selection of differentially expressed genes also likely reduced the noise in the dataset from spurious correlations between non-responding genes.

### Maize Jasmonate Related Network

Jasmonate signaling, and especially the jasmonate biosynthetic pathway have been implicated as a major determinant of resistance to fungal infection in multiple studies involving *Z. mays* and *A. flavus* (Lyons et al., 2013; Christensen et al., 2015; Magnin-Robert et al., 2015). This has also been demonstrated with multiple genes in *Arachis hypogaea* (Tsitsigiannis et al., 2005), *A. thaliana* (Pauwels and Goossens, 2011; Christensen et al., 2015), indicating some degree of conservation of these pathways in plants. Mining the GEN specifically for known genes in this pathway and identifying their neighboring genes (those genes strongly co-expressed with individual genes in this pathway) therefore provide one approach to discover new candidate genes involved in fungal resistance in *Z. mays*. The jasmonate pathway biosynthesis genes including OPR1 (GRMZM2G106303; 12-oxophytodienoic acid reductase), OPR2 (GRMZM2G000236; 12-oxophytodienoate reductase 2) and LOX (GRMZM2G156861_P01; Lipoxygenase) were all in the same co-expressed cluster of 446 *A. flavus* and *Z. mays* genes when the genes were clustered into 10 bins. When 100 bins (**Supplementary Table S2**) were used in the analysis, OPR1 and LOX5 (GRMZM2G102760; Lipoxygenase 5) occurred in cluster 39 and OPR2, LOX2 (GRMZM2G156861; Lipoxygenase 2) and PRMS (AC205274.3_FGP001; Pathogenesis-related protein) occurred in cluster 65.

In the co-expression network, OPR2 and OPR1 were found to be connected (tightly co-expressed) to six *A. flavus* genes, and one *Z. mays* gene (GRMZM2G371167) that is annotated only for “a response to low sulfur.” The *A. flavus* genes were (AFLA_061840) Xaa-Pro aminopeptidase P, Bax inhibitor (AFLA_061840), BZIP transcription factor (Bzip) (AFLA_083100), 4-nitrophenylphosphatase (AFLA_083100), Endo-1, 4-beta-xylanase A, putative (AFLA_083100), and Mitochondrial translation initiation factor IF-2 (AFLA_028660). OPR2 gene expression was not consistently up-regulated in all time points, but showed a complex pattern of induction and suppression over time after infection. Significant correlations were found for (LOX), SSP2 (GRMZM2G097641). The *A. flavus* genes autophagy cysteine endopeptidase Atg4 was found to be co-regulated with OPR2.

The COB co-expression network includes more than 100 experimental conditions and tissues; the network is typical of a global comprehensive expression correlation map, but notably does not include fungal pathogen experiments. When OPR2 was analyzed in the COB online co-expression network for *Z. mays*, no genes were found to be co-expressed with it Schaefer et al. (2014). Thus, the co-expression and clustering of genes shown in this study, including those involving OPR2, may reflect a specific response to fungal infection.

### Maize Ethylene Related Genes

Ethylene signaling and biosynthetic pathways were also significantly up-regulated in response to fungal pathogenesis, and many genes in those pathways were found to be co-expressed in the co-expression network with novel genes, or genes previously not associated with disease resistance. One of the ethylene genes found in the co-expression network, the ethylene signaling gene *EREB129*, an AP2ERE element binding protein-transcription factor (GRMZM2G016434), was found to have different connections (representing tight co-expression) within the network; it was linked with avr9/C-9 (GRMZM2G172695) an avirulence protein which has been shown to be involved in pathogen resistance studies involving *A. flavus*, *Cladosporium fulvum* (Chen et al., 2004; Rowland et al., 2005; Luo et al., 2008, 2011). *EREB108* was also found to be connected to (GRMZM2G137802), a WRKY7 transcription factor (GRMZM2G154828) a cytochrome P450 putative expressed gene, and to (GRMZM2G175076) a chalcone-flavanone isomerase family protein.

The ethylene-responsive transcription factor GRMZM2G474326 had 13 connections with multiple *Z. mays* genes including two reactive oxygen producing genes; (GRMZM2G087875) cytochrome P450, and AOX2, an alternative oxidase gene which had been implicated as being important in disease resistance by other studies (HanQing et al., 2010). Additionally, GRMZM2G474326 was found to be connected to (GRMZM2G104626) 3-ketoacyl-CoA synthase, and (GRMZM2G086869) haloacid dehalogenase-like hydrolase (HAD) superfamily protein. To our knowledge, these genes have not been previously implicated in disease resistance. Some of the other genes that were found to be linked to ethylene signaling related genes in this study, were experimentally determined to be involved in resistance to *Fusarium graminearum* (Hart et al., 2015).

### *A. flavus* Transport Genes

In *A. flavus*, a complex transport system is involved in secondary metabolism (Linz et al., 2011; Amare and Keller, 2014; Rohlfs, 2015). Specialized vesicles, aflatoxisomes, have been identified and were found to be involved in the transport of aflatoxin and other secondary metabolites (Chanda et al., 2010; Roze et al., 2011). Multiple vesicular transport genes were detected among the genes induced after inoculation within the GEN for *A. flavus.* These were genes that are conserved across eukaryotes, including the SNARE protein Snc2 (AFLA_016950), Vacuolar protein sorting associated protein vps17 (AFLA_057210) and many others (**Supplementary Table S1**).

### Interspecies Networks of Gene Expression Correlation

**FIGURE 3:**
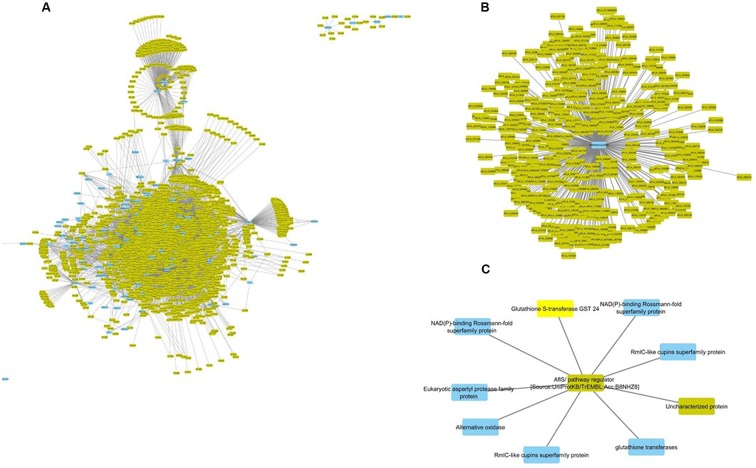
**Interspecific *Z. mays* and *A. flavus* co-expression network Pearson correlation analysis was performed on *Z. mays* and *A. flavus* genes to generate a cytoscape network.** The network contains edges (lines implying linkage) which had a Pearson correlation >0.95. Blue nodes (*Z. mays*) and yellow nodes (*A. flavus*) are found within the network. Within the network **(A)** represents the *Z. mays* genes that were found to be co-expressed with at least one *A. flavus* gene. **(B)** (GRMZM2G420988) Solute Carrier Protein which was found to be associated with 421 *A. flavus* genes. **(C)** Shows a subnetwork with the co-regulator for the aflatoxin cluster *AflS.*

One of the advantages of using GENs is the ability to identify interspecific gene expression correlations. The GEN was mined for interactions between *A. flavus* and *Z. mays*. This yielded a subnetwork of 2046 genes comprised of 102 *Z. mays* genes and 1944 *A. flavus* genes, and 9450 co-expression edges that crossed species (**Figure 3A**). Functional analysis was conducted on all 2046 genes. The 102 *Z. mays* genes were found to be involved in biological processes such as oxidation reduction (GO:0055114), oxidoreductase activity (GO:0016491), hydrolase activity (GO:0016787). One of the *Z. mays* genes, GST7, was connected with 776 *A. flavus* genes. The 776 *A. flavus* genes fell into different significant GO-terms including GO:0048193 (Golgi Vesicle Transport), and (GO:0030163) protein catabolic process (**Figure 3B**; Klionsky et al., 1990). An absence of maize intraspecific correlation was observed when these 102 genes were mined using the COB co-expression network (Schaefer et al., 2014). In *A. flavus*, the 1944 genes in the sub-network included genes involved in trehalose-related metabolic processes, transferase activity, and L-amino acid peptides-related peptidase activity.

The interspecies sub-network additionally revealed that many known resistance genes in maize are co-expressed with *A. flavus* genes. For example, the *Z. mays* pathogen resistance-5 gene (PR-5; GRMZM2G402631), which has been shown to be involved in systemic acquired resistance, was found to be correlated with AFLA_002560 (60S ribosomal protein) (Gómez-Ariza et al., 2007; Lanubile et al., 2015; Shu et al., 2015). This particular *A. flavus* ribosomal protein had previously been shown to be inhibited by antifungal activity (Delgado et al., 2015). Additionally, PR5 expression was correlated with 4303 *A. flavus* genes and 76 *Z. mays* genes that consisted of likely resistance gene candidates. The *Z. mays* gene included GRMZM2G039639, a Thaumatin family protein which has been shown to be transcriptionally up-regulated during host–pathogen interactions (Petre et al., 2011; Scarpari et al., 2014; Christensen et al., 2015). The three other additional partners where two cytochrome P450 and PR-1 which has also been implicated in resistance to *A. flavus* (Guo et al., 2008; Lanubile et al., 2015).

The most important finding of the interspecies sub-network was the presence of secondary metabolism genes related to aflatoxin biosynthesis. In fact, these genes formed a large interconnected module within the sub-network. One of the key regulatory genes of the aflatoxin biosynthesis cluster, AflS (AFLA_139340 formerly AflJ), had a dense connection of 1169 genes (nine maize, 1160 *A. flavus*). It was also found to be co-regulated with seven *Z. mays* reactive oxygen species (ROS) production genes including a Rmlc-like cupin protein, a glutathione *S*-transferase and an alternative oxidase 3 (**Figure 3C**). The expression for this secondary metabolism module of genes was highly similar, which is why it formed such a hub in the co-expression network. Many of these *Z. mays* genes co-expressed with AflS were involved specifically in the production of peroxides, which have been typically shown to be involved in the response of plants to abiotic and biotic stress (Mori and Schroeder, 2004; Torres et al., 2006). For example, Rmlc-like genes are involved in the production of peroxides via oxalate oxidase (Lane et al., 1993). Multiple germin-like proteins similar to these are known to be present within the *Z. mays* kernel tissue. The group of maize genes linked to *AflS* also included genes reported to be indicators of resistance (induced upon infection and associated with resistance in breeding studies) such as AOX, an alternative oxidase (Simons et al., 1999; Fung et al., 2004).

*AflS* was also found to be co-regulated with 1162 *A. flavus* genes in the network; the aflatoxin cluster genes *AflX*, *AflE*, *AflW*, *AflV*, *AflK*, *AflL*, *AflN*, *AflM*, *AflH*, *AflS*, *AflA*, *AflD*, and *AflC* were all found to be co-expressed with *AflS*. *AflS* was also found to be co-regulated with *A. flavus* secretion and transport protein encoding genes including the endomembrane trafficking proteins COPI vesicle (AFLA_044000), COPII vesicle (AFLA_030110) and the Golgi to endosome transport protein Ent3 (AFLA_092060). This agrees with a previous report of *AflS* being found to interact with multiple *A. flavus* genes involved in transport and development (Ehrlich et al., 2012). It is to be noted that *AflR*, a known regulator of the aflatoxin biosynthesis cluster, was absent in our network. This is mainly due to its exclusion from the network since it did not meet the cut-off criteria used in the initial filtering of the differentially expressed genes (see Materials and Methods). Similarly, several *A. flavus* genes from the aflatoxin biosynthesis cluster were not present in the network.

### Network Analysis

One of the key advantages of working with GENs over utilizing a traditional clustering approach, is exploiting the ability of graph theory to assess genes of importance. A key metric, connectivity, which is a measure of how many genes a given gene is co-expressed with, can also be used to infer possible biological connections including physical interactions and shared common regulators. The connectivity of some of the genes in the GEN is shown in **Table 1**. This type of connectivity has been demonstrated in multiple interactomes, co-expression networks for humans (*Homo sapiens*), *A. thaliana* and *Z. mays* due to the overlap between interactomes and co-expression data (Wachi et al., 2005; Gu et al., 2011; Musungu et al., 2015). Among *Z. mays* genes, several ROS genes were found to have the largest number of connections in the network. For example, the *Z. mays* glutathione *S*-transferase 7 (GST7), which has been previously implicated in ROS (Mideros et al., 2014; Yin et al., 2014; Fountain et al., 2015), had 776 connections, while cytochrome P450 family 72 (GRMZM2G147752), also involved in oxidative stress, had 223 connections in the interspecies network. Interestingly, the top 20 most connected genes in the network were *A. flavus* genes (**Table 2**). This group of genes includes *A. flavus* genes involved in the biosynthesis of methionine, carbohydrate metabolism genes such SNF1, and vesicular transport gene. The presence of highly co-regulated transport genes is not very surprising since endomembrane transport is a highly coordinated process. In fact, transport genes are typically found to be tightly connected in networks such as in the interactomes of *Z. mays*, *S. cerevisiae*, and *A. thaliana* (Yu et al., 2008; De Bodt et al., 2009; Musungu et al., 2015).

**Table 1:** Connectivity for the interspecific co-expression sub-network.

Gene ID	Annotation	Connectivity
CADAFLAG00013514	Unknown	1153
GST7	Glutathione *S*-transferase	776
GRMZM2G099467	Gibberellin 20 oxidase 2	561
GRMZM2G143669	Oxidoreductase short chain dehydrogenase	487
GRMZM2G049930	RmlC-like cupins superfamily protein	464
GRMZM2G157298	(GSTL2) glutathione transferase lambda 2	462
GRMZM2G071390	Germin-like protein	461
GRMZM2G320786	(LAC14) laccase 14	458
GRMZM2G087875	Cytochrome P450 superfamily protein	423
GRMZM2G334181	Putative DUF26 domain family protein	422
GRMZM2G420988	mitochondrial carrier protein putative expressed	421
GRMZM2G093092	*O*-methyltransferase family protein	354
AOX3	Alternative oxidase	332
GRMZM2G170017	Carbonyl reductase 1	272
GRMZM2G147752	(CYP72A14) cytochrome P450 family 72	233
GRMZM2G036365	Eukaryotic aspartyl protease family protein	205
GRMZM2G020631	NAD(P)-linked oxidoreductase superfamily protein	157
GRMZM2G148355	NAD(P)-binding Rossmann-fold superfamily protein	152
GRMZM2G466563	Calmodulin-binding protein	144
GRMZM2G039009	*S*-adenosyl-L-methionine-dependent methyltransferases	137

**Table 2:** Connectivity of the top 20 most connected genes in the gene co-expression network.

Gene ID	Count	Annotation
AFLA_003910	1572	Nuclear condensin complex subunit 3, putative
AFLA_055600	1568	1,2-dihydroxy-3-keto-5-methylthiopentene dioxygenase
AFLA_090380	1562	Chromatin remodeling complex subunit (Arp9), putative
AFLA_103750	1554	Phospholipase D1 (PLD1), putative
AFLA_022590	1554	YdiU domain protein
AFLA_080160	1553	UDP-*N*-acetylglucosaminyltransferase
AFLA_016890	1553	Rho GTPase activator (Lrg11), putative
AFLA_061950	1548	Autophagy-related protein 3
AFLA_051550	1547	Methylthioribulose-1-phosphate dehydratase
AFLA_027290	1544	Proteasome activator subunit 4, putative
AFLA_133590	1539	Cell polarity protein (Tea1), putative
AFLA_020340	1538	Sulfate transporter, putative
AFLA_027140	1535	ADAM family of metalloprotease ADM-B
AFLA_111310	1534	Monosaccharide-*P*-dolichol utilization protein, putative
AFLA_021530	1534	Putative uncharacterized protein
AFLA_127460	1531	SNF7 family protein
AFLA_130150	1530	NAD+ dependent glutamate dehydrogenase, putative
AFLA_103810	1529	Spindle poison sensitivity protein Scp3, putative
AFLA_041020	1529	Putative uncharacterized protein

Further analysis of the GEN with the network analyzer module in Cytoscape revealed that the characteristic path lengths of the network was 2.773, which was smaller than the previously reported co-expression network for *A. thaliana* (5.065) (Cline et al., 2007; Carrera et al., 2009). This is likely due to the approach we used to select the input genes from the initial analysis of the network. In similar work described in the literature, all possible genes, as opposed to a select few, are sometimes used to generate co-expression networks (Schaefer et al., 2014). Our network, however, had a larger clustering coefficient of 0.606 in comparison to the reported 0.309 clustering coefficient in the *Arabidopsis* network.

### Module Discovery of the Gene Co-expression Network Utilizing MCODE

**FIGURE 4:**
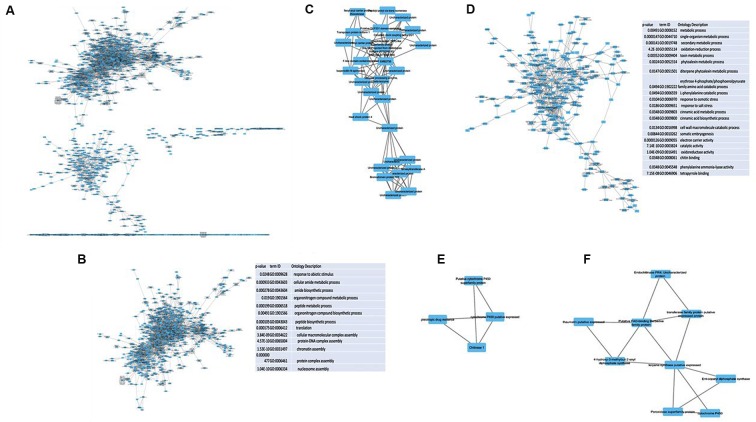
**(A)** Mcode Analysis of the *Z. mays* to *Z. mays* subnetwork from the gene co-expression network (GEN) was performed. **(B,D)** Shows subnetworks and the functionally enriched terms present within each of subnets that had greater than two interactions. **(C,E)** Show the genes that had the largest clustering coefficient in the subnetworks. **(C)** Represents the mcode analysis of **(B,E,F)** represents **(D)**. For the tables show expression of the columns were order by a relative to the abundance of *A. flavus*. The gene ontology values were calculated using gprofiler and only significant terms were kept in the table (FDR <0.1).

MCODE clustering analysis identifies highly interconnected subgraphs within the co-expression network to determine significant clusters (Bader and Hogue, 2003). Initial analysis of our network identified multiple modules specific to *Z. mays- Z. mays* that were either coordinately up-regulated or down-regulated (**Figure 4A**). A large number of the gene expression patterns in *Z. mays* and *A. flavus* are oscillatory in nature due, among other things, to the circadian rhythm. The network was split into two subnets as shown in (**Figures 4B,C**). In one of the down-regulated modules, a set of differentially expressed genes of *Z. mays* included carrier proteins, protein biosynthesis genes, and response to abiotic stress genes. Significant clusters determined by MCODE clustering coefficient had functions that were related to circadian rhythm and vacuolar processes and were down-regulated through all of the samples (**Figure 4C**). Surprisingly, a number of heat shock proteins were also found to be significantly downregulated in these clusters; these genes are typically up-regulated during biotic stresses (**Figure 4C**; Chen et al., 2002; Jones and Dangl, 2006).

Multiple genes related to resistance to pathogens are visualized in one of the subgraphs featuring up-regulated genes (**Figure 4D**). These genes were highly enriched for fungal specific responses, including the production of phytoalexins and chitin sensing, as well as for abiotic stress response. This sub-network seems to represent an up-regulated defense module. **Figure 4E** displays a subgraph with 4 *Z. mays* genes including (GRMZM2G358153) chitinase 1, GRMZM2G161472 (cytochrome P450), GRMZM2G122654 (cytochrome P450) and GRMZM2G415529 (pleiotropic drug resistance 11). The chitinase 1 gene has been often shown to be involved in resistance to pathogens. Moreover, MCODE pulled a gene subnetwork with a nine gene module believed to be related to pathogen resistance (**Figure 4F**). The module included (GRMZM2G129189) Endochitinase PR-4, which has been associated to the resistance to *A. flavus* and to other ear rots causing fungi (Wang et al., 1996; Borad and Sriram, 2008; Sels et al., 2008; Fountain et al., 2010).

Similar steps were taken to identify highly interconnected subgraphs within the *A. flavus-A. flavus* network. One module contains the aflatoxin cluster genes, including *O*-methyltransferase (AFLA_120990), *O*-methylsterigmatocytsin oxidoreductase (AFLA_139200), Noranthrone monoxygenase (AFLA_081900) as well as a previously uncharacterized gene (AFLA_081900) which may represent a new gene associated with the aflatoxin biosynthesis pathway.

### Interactome Overlap

**FIGURE 5:**
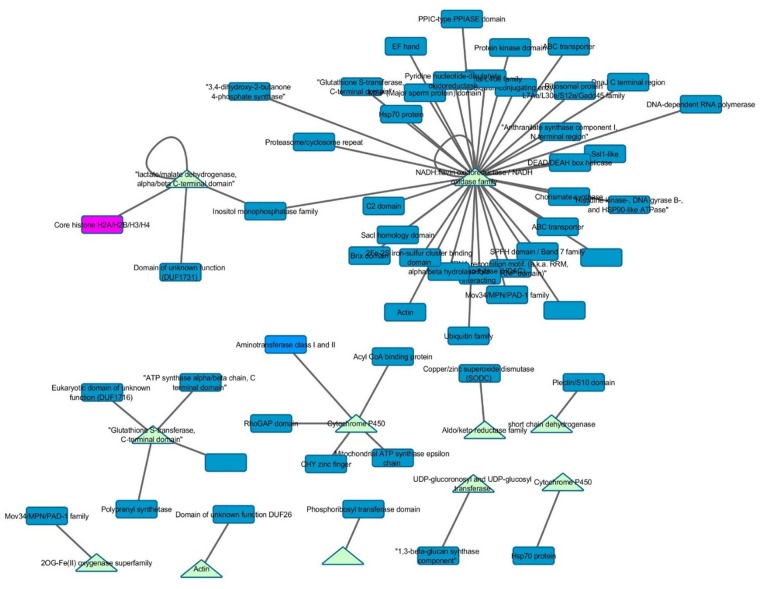
**Gene co-expression and interactome overlap network.** Genes found in the interspecies co-expression network (baits) were mined in the predicted maize interactome (PiZeaM) to determine novel interacting protein partners (preys). Triangles indicate that a gene is a bait gene from the static co-expression network and rectangles indicate first neighbors within the interactome.

Interactomes have been noted to overlap with gene co-expression, presumably due to the selection toward improved efficiency in forming pathways and protein complexes (Bhardwaj and Lu, 2005). Therefore, *Z. mays* genes that were present within the GEN were mined for any potential physical connections in the maize interactome PiZeaM (Musungu et al., 2015). Of the 102 *Z. mays* genes that were found in the interspecies GEN (**Figure 3**), a small subnetwork of interacting proteins and first neighbors was identified. None of the *Z. mays* genes in that protein interactome subnet were significantly co-expressed in the GEN. Many of these proteins were involved in defense specific responses, such as oxidative stress pathway and jasmonic acid pathway, and thus may represent plant and maize specific patterns of co-expression not likely to be identified using the interlog method that produced the maize interactome. In the first neighbor analysis of the interactome proteins, we found multiple resistance genes that can be utilized for further investigation, such as OPR2. The interactome subnetwork analysis revealed that OPR2 was the largest hub, and that it interacted with heat shock proteins, glutathione proteins and PAD-1 domain containing proteins. Some of these genes have been previously implicated in resistance (**Figure 5**; Ferrari et al., 2003).

## Discussion

This work represents the first cross species co-expression network study of maize and *A. flavus*, and is also the first *A. flavus* co-expression network study to our knowledge. Our approach was taken to allow the mining of the data for non-traditional information in order to determine significant pathways that are correlated in *A. flavus* and *Z. mays*. The GENs are beneficial tools that are able to address questions dealing with complex biological systems. Because of the benefits of these types of analyses, RNA-seq gene expression data from an *A. flavus*/*Z. mays* time course experiment was used to identify novel genes of interest that are involved in this interaction. Initial analysis of the network demonstrated key properties that are shared with co-expression studies such as the high density of edges produced from the data set. One of the benefits from the gene co-expression analysis is the ability to infer the functional characteristics of a group of interacting genes. It also permits the analysis of genes that can otherwise be lost when doing QTL analysis for polygenic resistance studies. When combining these benefits with systems biology, it is possible to build networks of interest that allow for the use of guilt by association to infer possible common regulators of co-expressed genes and pathways. Moreover, GENs allow the use of *a priori* information to mine for new hypotheses.

In this analysis, novel genes identified by clustering and network analysis may be involved in aflatoxin production and transport, maize resistance to pathogens, and possibly host recognition. The central importance of jasmonate, ethylene, and ROS is clearly evident on the maize side, as is the co-expression linkage between pathogen toxin production and transport. Gene correlations were also detected across species. This subnetwork was highly asymmetric, with only 102 maize genes and 1944 pathogen genes. For the 102 maize genes that were found in the co-expressed network, it was determined that their functions were highly related to resistance to pathogens. This was seen by the presence of genes such as PR1and PR5. This is highly unusual, as roughly equal numbers of maize and A. *flavus* genes were differentially expressed, thus equivalent numbers of genes for host and pathogen co-expression would be expected by random chance. That *A. flavus* had almost 20 fold more genes than maize that responded across species may indicate that the pathogen was closely monitoring and responding to the host through these 102 key maize genes. These “pathogen” genes may thus represent part of the host recognition and response gene pathways.

One of the beneficial aspects of GENs is the ability to use them to complement the data generated from differentially expressed genes studies. One of the interesting findings in this study that correlates with previously reported research is the importance of *Z. mays* genes, such as LOX4, LOX6, OPR1, OPR2, in the *A. flavus/Z. mays* interaction. In this study, these genes were captured in the GEN. Also identified in the GEN were *A. flavus* genes related to transport signaling, such as COPI and COPII, as well as the ROS production genes in *Z. mays* AOX, peroxidase, and glutathione *S*-transferase. This matches previous studies reporting that mutations in lipoxygenases in *Z. mays* can have downstream effects on disease development and on aflatoxin production (Burow et al., 1997; Park et al., 2010; Scarpari et al., 2014; Throckmorton et al., 2016). Additionally, the GEN developed in this study revealed that several genes involved in ethylene induction are correlated with multiple *A. flavus* genes, exposing another facet of the interaction between *A. flavus* and *Z. mays*. This correlation could reflect one of the mechanisms *A. flavus* employs to respond to *Z. mays* ROS producing enzymes.

To fully exploit an *A. flavus* predicted protein–protein interactome (SUBEDI et al., 2015) multiple clustering, and GEN analyses were used. The generated data complemented the co-expression analysis, and multiple *A. flavus* genes were found to play a significant role in the *A. flavus/Z. mays* interaction as revealed in GENs and by K-means clustering. In fact, a hub containing 128 genes of interest was found to be co-expressed with secondary metabolite genes and transport genes in *A. flavus*. Each one of these 128 genes was mined in the String database of *A. flavus* to determine which genes should be considered as candidates for further studies (Szklarczyk et al., 2015). Interestingly, the gene (AFLA_026380) UbiD/Ubi4 was a center hub in the network interacting with multiple vesicle, endosome, and carbohydrate metabolism genes. This gene also interacted with (AFLA_025590) PalF, a pH regulator, and (AFLA_049870) AreA, a nitrogen regulator. This interaction is a key one because it has not been reported in previous studies, and involves two key domains involved in pH and nitrogen metabolism (Ehrlich et al., 2005; Tudzynski, 2014). Moreover, UbiD, also ranked the highest by connectivity in the *A. flavus* interactome (PiAF; SUBEDI et al., 2015), which reflects the biological importance of this protein.

Another interesting result from this study is the revealed importance of ROS in both *A. flavus* and *Z. mays* during the early interaction between these two organisms. The generated GEN reveals an extensive co-regulation of *A. flavus* genes involved in the aflatoxin biosynthesis cluster involving and *Z. mays* ROS genes. For instance, *AflJ/S* was found to be co-regulated with multiple *Z. mays* resistance genes that are known to be involved in the production of peroxides. This is, to our knowledge, the first report demonstrating that this interaction may be occurring during the early interaction between *A. flavus* and *Z. mays.* It also reveals one of the potential roles of *AflJ*/*S* which probably has multiple functions as demonstrated by transformation studies with *AflJ/S* orthologs (Chettri et al., 2015). It is to be noted here that the majority of the previously reported studies investigating genes such as AP-1, AtfB, Hsf-2, Skn-7, and Msn2-4, which are known regulators of ROS, were performed in culture and therefore not necessarily reflective of what actually occurs during the interaction between a pathogen and its host (Reverberi et al., 2005, 2008, 2010).

## Conclusion

This work goes beyond the identification of genes relying primarily on differential expression of genes and uses GENs to infer functions of interest in *A. flavus* and *Z. mays*. Novel information from this study shows that *A. flavus* utilizes different mechanisms in response to the induction of resistance genes in *Z. mays* during the early interaction between the two organisms.

## Accession Number

NCBI Accession Number SRP082421.

## Author Contributions

Field work and RNA extraction was done by GO and GP. The data was analyzed by BM and MG. The manuscript was written and edited with BM, DP, AF, GP, and RB.

## Conflict of Interest Statement

The authors declare that the research was conducted in the absence of any commercial or financial relationships that could be construed as a potential conflict of interest.

## Supplementary Material

FIGURE S1 **A random matrix of counts was converted to M-values (see Materials and Methods) and Pearson correlation values were calculated.** The random correlation values are represented by the light blue color and yellow represents the differentially expressed correlation values for *Aspergillus flavus* and *Zea mays.* Additionally to determine if the distributions were significantly different a Kolmogorov–Smirnov test two sided was performed and significant *p*-value 2.2e × 10^–16^, *D* = 0.40356.

TABLE S1 **Gene expression matrix for correlation analysis**.

TABLE S2 **Data from K-means clustering analysis**.
