# Supplementary material for: A Network Approach of Gene Co-expression in the Zea mays/Aspergillus flavus Pathosystem to Map Host/Pathogen Interaction Pathways
Source: Front Genet. 2016 Nov 21;7:206. doi: 10.3389/fgene.2016.00206 (PMC5116468; doi:10.3389/fgene.2016.00206)
Supplement: Supplementary file 1 [file Data_Sheet_1.DOCX]

This link contains access to the supplemental expression data hosted at gene express omnibus. It will also contain the CLC output files for the gene expression data.

**The following link has been created to allow review of record GSE85860 while it remains in private status:**

[**http://www.ncbi.nlm.nih.gov/geo/query/acc.cgi?token=crwvyiuuttollwf&acc=GSE85860**](http://www.ncbi.nlm.nih.gov/geo/query/acc.cgi?token=crwvyiuuttollwf&acc=GSE85860)

**Please note the following points:**

**- This link allows anonymous, read-only access to GSE85860 and associated accessions;**

**- You should send the link to the journal editor when you submit your manuscript -- the editor will circulate the link to reviewers requiring access to your private data;**

**- Do not include this link in your publication. For information on GEO linking and citing, please refer to** [**http://www.ncbi.nlm.nih.gov/geo/info/linking.html**](http://www.ncbi.nlm.nih.gov/geo/info/linking.html)**;**

**- Please contact us at** [**geo@ncbi.nlm.nih.gov**](mailto:geo@ncbi.nlm.nih.gov) **if you need to revoke access from this link**

**Regards,**

**The GEO Team**
