# Supplementary figures and images for: A Network Approach of Gene Co-expression in the Zea mays/Aspergillus flavus Pathosystem to Map Host/Pathogen Interaction Pathways
Source: Front Genet. 2016 Nov 21;7:206. doi: 10.3389/fgene.2016.00206 (PMC5116468; doi:10.3389/fgene.2016.00206)

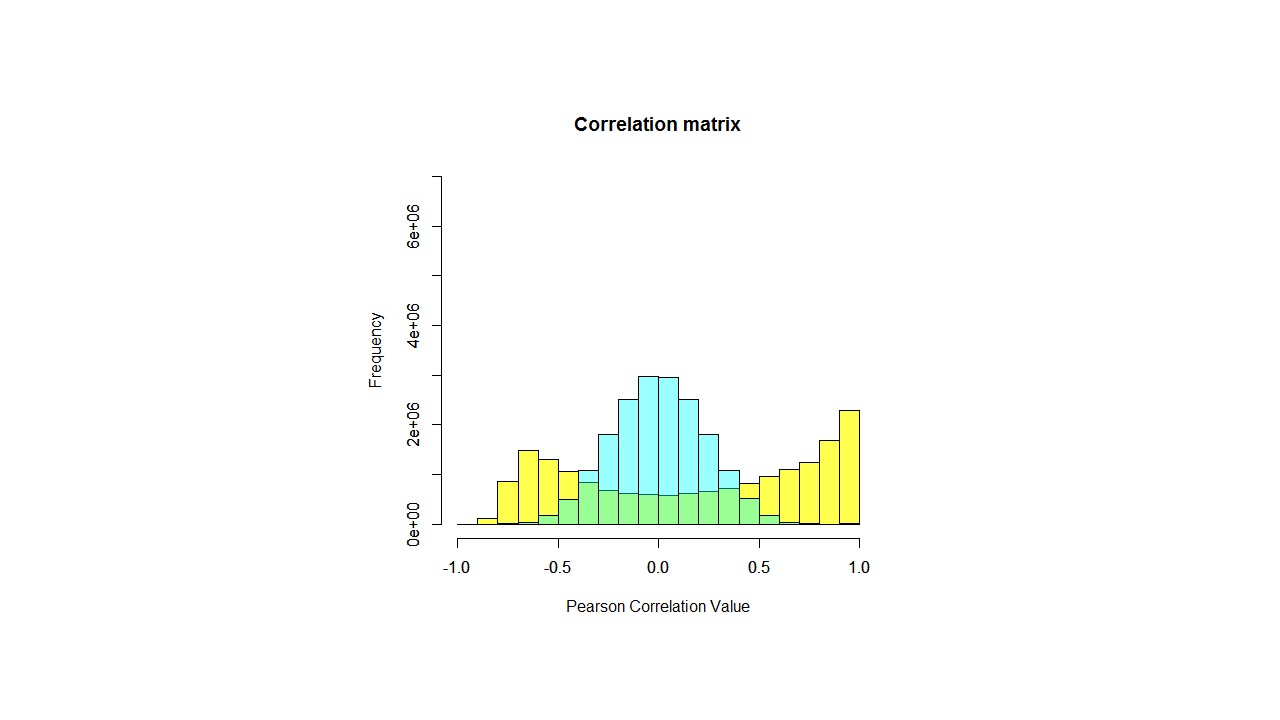

Supplement: FIGURE S1 — A random matrix of counts was converted to M-values (see Materials and Methods) and Pearson correlation values were calculated. The random correlation values are represented by the light blue color and yellow represents the differentially expressed correlation values for Aspergillus flavus and Zea mays. Additionally to determine if the distributions were significantly different a Kolmogorov–Smirnov test two sided was performed and significant p-value 2.2e × 10–16, D = 0.40356. [file Image_1.JPEG]
